# Vanity or Madness?

**Published:** 1888-09-22

**Authors:** 


### VANITY OR MADNESS?

One of the strangest phenomena of that often undesired resort of the diseased in soul, the police-court, is the sham confession of serious crimes. That a man who, in a moment of drunkenness or mental aberration, at some moment when the control ordinarily exercised by the moral law was weak, had committed a crime—theft, forgery, even murder—should avow his guilt when reason had returned and conscience had regained her sway is an infrequent, but not impossible occurrence. That any man should accuse himself unjustly of a deed of a criminal nature seems, on the face of it, incredible. Yet such things occur, and not so rarely as might be expected. The sham confession of murder is one of the psychical curiosities that exist, though not dreamed of in the philosophy of sanity or common sense. Only a week or two ago, a man was accused, on his own confession, of a murder committed seven years ago. As, by our —in this respect at least—sensible law, a man cannot be condemned on his own confession, the machinery of the police was set to investigate the truth of the statement, with the result that it was conclusively proved that the would-be murderer was innocently engaged in his daily work at the time the crime was committed, and thereore could not be guilty. He was dismissed, with a fretful complaint from the magistrate that he had wasted the time of the Court, though a female relative of the murdered man expressed her conviction in face of the ascertained facts that the accusation was true, and the legal disproof of it as well as the subsequent recantation of the self-accused totally false. What, it may be asked, would make any man risk life, or at least liberty, by accusing himself of such a crime? Madness only, is the instinctive reply; but in many men of the weaker sort there exists a morbid vanity, akin to madness, which yet is not identical with it. There are fire—drawing-room-fire-eating heroes who magnify their exploits; there are criminals who when safely imprisoned and interviewed by the irrepressible journalist multiply their burglaries with violence by ten; while there are legends, whether true or false we do not pretend to say, that if a moderately young woman has to stand her trial for the murder of husband or lover her attention to the serious issues at stake is distracted by offers of marriage from men she has never seen—men who evidently rank notoriety above honour, and seek it without inquiring too closely about its nature. From the ranks of these come the *soi-disant* murderers, who in real life, like the executioner in the *Mikado*, cannot kill anything—from these, or from a class more pitiable still, for whom, moreover, our pity need not be mingled with either anger or contempt. This is the class of the wearily unfortunate, to whom life and liberty have been so harsh that death or imprisonment seems preferable. Some of these go on tramp because they know that if they are away the parish will do something for their wives and children, and for themselves the scant fare and hard work of a casual ward are better than the enforced idleness and starvation they must otherwise endure. Others steal, and so get sent to prison for more or less lengthened periods; the most desperate accuse themselves of murder, knowing that the last penalty of the law is seldom exacted from those who voluntarily confess their past sins, and hoping for the shelter and comfort of lifelong imprisonment. From one or other of these classes are the self-accusers drawn, but, to whichever they belong, can it be right, because they have committed no crime, to let them go forth without any attempt at supervision being exercised over their distraught minds or despairing hearts? Some day they may be tempted to deserve the condemnation they now ask for.

